# Prevalence and Clinical Significance of Limb Arterial Variations: A Systematic Review and Proportional Meta-Analysis with an Evidence-Based Educational Framework

**DOI:** 10.3390/diagnostics16142163

**Published:** 2026-07-10

**Authors:** Andrei Nicolae Avadanei, Radu Florin Popa, Livia Genoveva Baroi, Cristina Strobescu-Ciobanu, Ionuț Mihai Cazan, Vladimir Țiței, Ștefana Avadanei-Luca, Sorin Nicolae Peiu, Anca Sava

**Affiliations:** 1 Department of Vascular Surgery, Grigore T. Popa University of Medicine and Pharmacy, 700115 Iași, Romania; andrei.avadanei@umfiasi.ro (A.N.A.); radu.popa@umfiasi.ro (R.F.P.); livia.baroi@umfiasi.ro (L.G.B.); cristina.c.strobescu-ciobanu@umfiasi.ro (C.S.-C.); ionut-mihai.cazan@d.umfiasi.ro (I.M.C.); vladimir.titei@d.umfiasi.ro (V.Ț.); 2 Department of Vascular Surgery, St. Spiridon Clinical Emergency Hospital, 700111 Iași, Romania; 3 Department of Plastic and Reconstructive Surgery, Grigore T. Popa University of Medicine and Pharmacy, 700115 Iași, Romania; stefana_luca@umfiasi.ro; 4 Department of Plastic and Reconstructive Surgery, St. Spiridon Clinical Emergency Hospital, 700111 Iași, Romania; 5 Department of Anatomy, Grigore T. Popa University of Medicine and Pharmacy, 700115 Iași, Romania; sava.anca@umfiasi.ro; 6 Department of Pathology, “Prof. Dr. N. Oblu” Clinical Emergency Hospital, 700309 Iași, Romania

**Keywords:** anatomical variation, limb arteries, vascular anatomy, CT angiography, proportional meta-analysis, Evidence-Based Anatomy, surgical education, patient safety

## Abstract

**Background/Objectives:** Anatomical variation of the limb arteries is a recurrent threat to safe surgical and interventional practice, yet it is frequently under-emphasized in modern curricula. **Methods:** We conducted a systematic review and proportional meta-analysis, following the PRISMA 2020 and Evidence-Based Anatomy methodology, to generate pooled prevalence estimates for the principal limb arterial variants and translate them into an educational framework. Six databases were searched from inception to December 2024; two reviewers independently assessed eligibility and methodological quality (AQUA tool); and proportions were pooled using the Freeman–Tukey double arcsine transformation under DerSimonian–Laird random-effects models. **Results:** Of 2847 records, 57 studies (23,267 limbs) were included. Pooled prevalence was 11.1% (95% CI 9.2–13.1) for high-origin radial artery, 2.6% (1.9–3.3) for superficial ulnar artery, 12.2% (6.5–19.2) for persistent median artery, 10.4% (9.1–11.9) for popliteal branching variants, and 0.8% (0.3–1.5) for peronea magna. Variation thus affects roughly one in five upper limbs and one in ten lower limbs—common enough to warrant deliberate curricular attention. **Conclusions:** We propose a four-principle framework (embryological reasoning, clinical-consequence integration, systematic identification, and competency-based assessment) for embedding variation recognition across the surgical training continuum.

## 1. Introduction

Anatomical variation poses a recurring threat to safe surgical and interventional practice. The typical brachial bifurcation at the cubital fossa, the textbook tibial-popliteal trifurcation, and the canonical appearance of the median artery as a vestigial structure are taught as norms; the morphological literature, however, demonstrates substantial deviation from these patterns in clinically meaningful proportions of individuals [1,2,3]. Time pressure on undergraduate anatomy instruction has compounded the problem: dissection hours have shrunk, and arterial variations are increasingly relegated to footnotes that students may never connect to the operating room or interventional suite [4].

The patient-safety stakes are well documented: misidentified upper-limb arteries can lead to inadvertent intra-arterial drug injection with limb loss [5], and an unrecognized peronea magna has caused devascularising complications after fibula free flap harvest [6]. The quantitative implications of these complications for clinical practice are returned to in the Discussion.

Anatomical variation can be conceptualized as a threshold concept [7]: a transformative idea that, once internalized, fundamentally restructures how trainees integrate anatomy with embryology and clinical practice. Recognizing variation as the morphological expression of developmental channel selection, rather than as a list of curiosities, allows trainees to reason across systems and to anticipate the unfamiliar. Translating this insight into curricular practice, however, requires evidence on prevalence, consequences, and educational design that the current literature has not synthesized in one place.

We therefore conducted a systematic review and proportional meta-analysis of limb arterial variations specifically framed for surgical educators. Our objectives were to (1) generate pooled prevalence estimates for the principal upper- and lower-limb arterial variants; (2) summarize the embryological and clinical evidence relevant to each variant; and (3) develop an evidence-based educational framework for embedding variation into competency-based surgical training across undergraduate, graduate, and fellowship-level curricula. Whereas previous proportional meta-analyses in this field have characterised individual arterial variants in isolation (for example, single-variant syntheses of the radial artery, the cerebral arteries, or the coronary arteries), the present review intentionally synthesises six clinically important upper- and lower-limb arterial variants in a single paper. We note that prevalence is not pooled across variants; each variant has its own separate proportional meta-analysis with its own k, pooled estimate, and heterogeneity statistics. The single-paper format is required by our educational purpose: a curricular framework that links variation recognition to surgical training across both upper and lower limbs needs a quantitative evidence base that spans the variants the trainee will actually encounter. Presenting these syntheses together also permits cross-variant comparison of detection-methodology effects, regional distribution, and the relative contribution of each variant to overall procedural risk—comparisons that are not available from single-variant reviews.

## 2. Materials and Methods

### 2.1. Study Design and Reporting

We followed Evidence-Based Anatomy methodology [8] and reported the review according to the PRISMA 2020 statement [9]. The protocol (eligibility criteria, search strategy, extraction template, quality assessment and statistical plan) was developed a priori. The complete search strategy is provided as Supplementary Appendix S1.

### 2.2. Eligibility Criteria

Eligibility was specified using a PICOS framework. Population: human subjects with documented arterial anatomy of the upper or lower limb. Comparator: typical arterial configuration. Outcomes: prevalence of arterial variations, morphological characterization, and reported clinical consequences. Study designs: cadaveric dissection studies, imaging-based cohort studies (conventional angiography, CT angiography, MR angiography, ultrasonography) and surgical case series. We excluded studies reporting exclusively on acquired vascular pathology, reviews without original data, conference abstracts without retrievable full text, samples below three specimens, and reports with inadequate methodological description.

### 2.3. Information Sources and Search Strategy

We searched PubMed/MEDLINE, Scopus, Web of Science, Embase, CINAHL, and Google Scholar from database inception through December 2024 with no language or date restrictions. Search terms combined controlled vocabulary and free-text strings. Reference lists of all included studies and three relevant systematic reviews were hand-searched, and forward citations of three landmark studies [1,2,3] were tracked. An academic librarian was consulted to refine the search. The primary electronic database search was completed in December 2024; three eligible studies that appeared online ahead of print after this date [10,11,12] were identified through forward citation tracking of three landmark references [1,2,3] and through hand-searching of journal indices during full-text extraction and manuscript preparation. No additional database searches were conducted after December 2024.

### 2.4. Study Selection and Data Extraction

Records were de-duplicated and screened in reference management software. Title and abstract screening was performed by one reviewer (A.N.A.) with uncertain records adjudicated against a second reviewer (Ș.A.-L.). Full-text eligibility was assessed independently by two reviewers (A.N.A. and R.F.P.); disagreements were resolved by discussion, with a third reviewer (A.S.) consulted when consensus was not reached. Inter-rater agreement was quantified using percent agreement and Cohen’s κ, interpreted per Landis and Koch [13]. Data were extracted by the primary reviewer and verified by a second reviewer (Ș.A.-L.). Extracted variables included study identifiers, design, detection method, sample size in limbs, variant type, count of variants, prevalence, and reported clinical consequences.

### 2.5. Quality Assessment

Methodological quality was assessed independently by two reviewers using the Anatomical Quality Assessment (AQUA) tool [14] across its five domains. Inter-rater agreement was quantified with Cohen’s κ. All eligible studies were included in the primary meta-analysis regardless of quality rating; the robustness of pooled estimates was probed in sensitivity analyses excluding studies classified as low-quality.

### 2.6. Statistical Analysis

Proportional meta-analyses used the Freeman–Tukey double arcsine transformation to stabilize variance [15] under a DerSimonian–Laird random-effects model specified a priori. For each variant we estimated pooled prevalence with 95% confidence intervals (CIs) and prediction intervals. Heterogeneity was assessed with Cochran’s Q (significance set at *p* < 0.10) and the I^2^ statistic, interpreted as low (≤25%), moderate (26–75%), or high (>75%) [16]. Sensitivity analyses excluded studies rated as low-quality; small-study effects were assessed using Egger’s regression where k ≥ 10 [17]. The persistent sciatic artery, with an estimated prevalence of 0.03–0.06%, was reported narratively rather than meta-analytically. The Freeman–Tukey/DerSimonian–Laird approach was retained as the primary analysis because it stabilizes the variance of proportions across the wide prevalence range covered by the included variants (including the rare-event range relevant to peronea magna), avoids the convergence and small-strata problems encountered with generalized linear mixed-effects models when several syntheses contain fewer than ten contributing studies, and permits direct comparability with the existing Evidence-Based Anatomy literature [8]. Where the primary synthesis exhibited substantial heterogeneity (I^2^ > 50%), exploratory subgroup analyses by detection modality (cadaveric dissection versus imaging-based studies), continent of study origin, and publication period were performed when at least four studies contributed within each subgroup level; these are interpreted as hypothesis-generating rather than confirmatory. Following current methodological recommendations from the Evidence-Based Anatomy guidelines for proportional (prevalence) meta-analyses, we additionally assessed small-study effects using a Doi plot with the LFK (Luis Furuya-Kanamori) index [18], which is less prone to bias than Egger’s regression in the prevalence setting. The LFK index was interpreted as no asymmetry (|LFK| ≤ 1), minor asymmetry (1 < |LFK| ≤ 2), or major asymmetry (|LFK| > 2). Pre-specified subgroup analyses by detection methodology (cadaveric dissection versus imaging-based studies) and by geographic region (continent) were performed for each variant where at least two contributing studies were available within each subgroup level; subgroup pooled prevalence was computed under the same Freeman-Tukey/DerSimonian-Laird framework and is reported in Table 2. All statistical analyses were performed in R (version 4.3.2, R Foundation for Statistical Computing, Vienna, Austria) using the meta (version 6.5) and metafor (version 4.6) packages; records were managed, de-duplicated, and screened in Zotero (version 6.0, Corporation for Digital Scholarship, Vienna, VA, USA).

### 2.7. Educational Analysis Framework

To derive the educational framework, all 57 included studies were re-read for educational relevance using inductive thematic coding (A.N.A. and Ş.A.-L.). Each study was coded against four candidate dimensions specified a priori from the Evidence-Based Anatomy and surgical-education literature: (i) embryological explanation, (ii) clinical consequence, (iii) teaching recommendation, and (iv) assessment implication. Coders worked independently; coding agreement was 87.4% at the first pass, and the remaining discrepancies (n = 28 study dimension assignments) were resolved by line-by-line discussion until full consensus was reached. After consensus, the four candidate dimensions were re-tested against the coded data and emerged unchanged as the framework’s four organizing principles. The distribution of studies contributing evidence to each principle was: embryological explanation (n = 41), clinical consequence (n = 49), teaching recommendation (n = 22), and assessment implication (n = 11). Mapping to the surgical training continuum (UME pre-clinical, UME clinical/clerkship, GME residency, GME fellowship) was performed by consensus discussion among authors with educational responsibilities at each level. The framework is presented as an evidence-informed proposal rather than as an empirically validated curricular intervention; controlled educational outcome studies remain a future-research priority (Section 5.2).

## 3. Results

### 3.1. Study Selection and Characteristics

**Figure 1:**
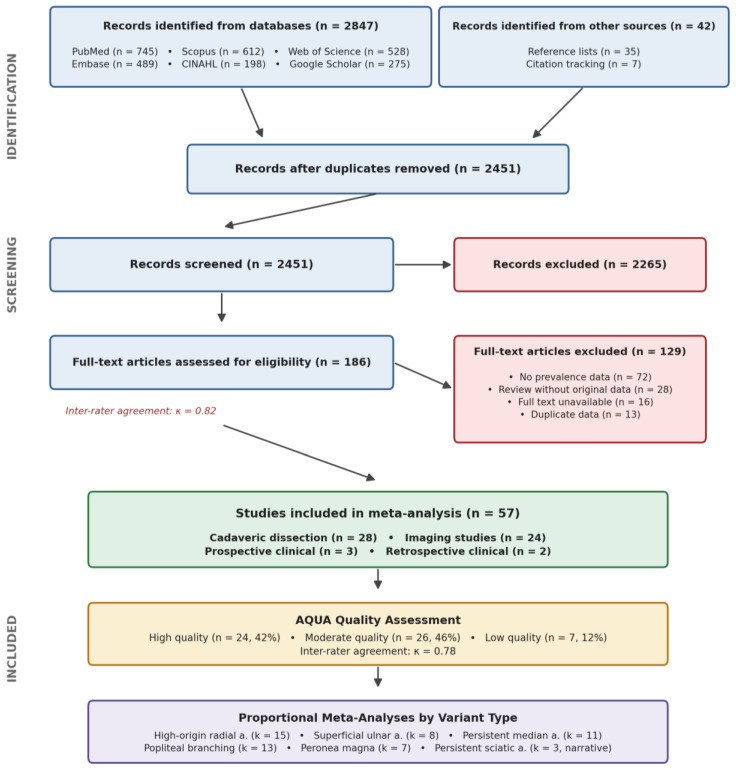
PRISMA 2020 flow diagram illustrating the study selection process.

Database searching identified 2847 records, with a further 42 records from reference lists and citation tracking. After removing 438 duplicates, 2451 records underwent title and abstract screening. Following full-text review of 186 articles, 57 studies meeting inclusion criteria were retained, providing prevalence data on six variant types and encompassing 23,267 limbs (Figure 1). Inter-rater agreement for full-text eligibility was substantial (κ = 0.82; percent agreement 91.4%). Included studies comprised cadaveric dissection (49%), imaging-based cohorts (42%), and surgical series (9%); geographic distribution spanned Europe (33%), Asia (26%), North America (23%), Africa (9%), South America (5%), and Oceania (4%). Complete study characteristics are provided in Supplementary Table S1. The funnel from 2847 identified records to 57 included studies (approximately 2%) reflects the breadth of the initial search strategy (intentionally broad to maximize sensitivity), the high proportion of records reporting on acquired vascular pathology, non-arterial structures, or non-limb territories, and the strict eligibility criteria applied for original prevalence data on the targeted variants (Section 2.2).

### 3.2. Quality Assessment

AQUA (inter-rater κ = 0.78; percent agreement 88.5%) classified 24 studies (42%) as high-quality, 26 (46%) as moderate, and 7 (12%) as low-quality. The low-quality stratum was dominated by historical publications (pre-1960) with limited reporting of specimen-selection criteria and dissection methodology. All studies were retained in the primary pool; the effect of exclusion is presented in sensitivity analyses below.

### 3.3. Upper-Limb Arterial Variations

**Figure 2:**
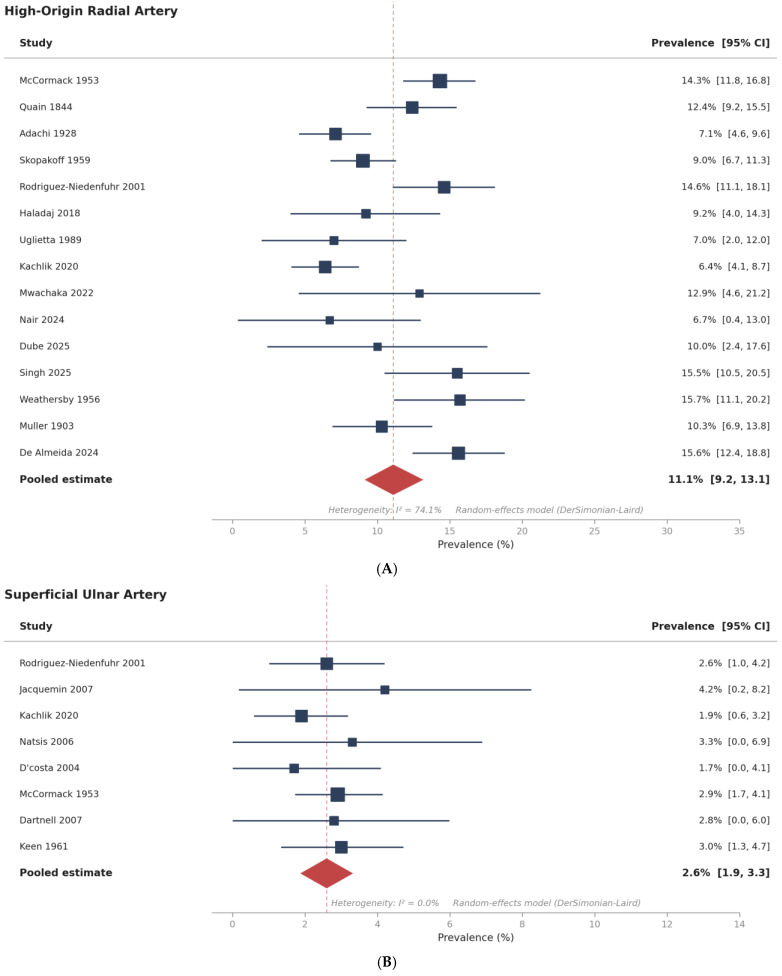

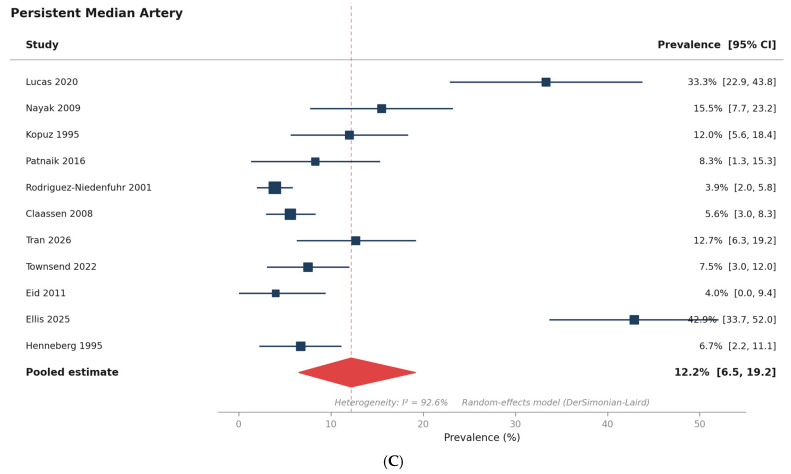
Forest plots of proportional meta-analysis for upper-limb arterial variations: (**A**) high-origin radial artery; (**B**) superficial ulnar artery; (**C**) persistent median artery.

**Figure 3:**
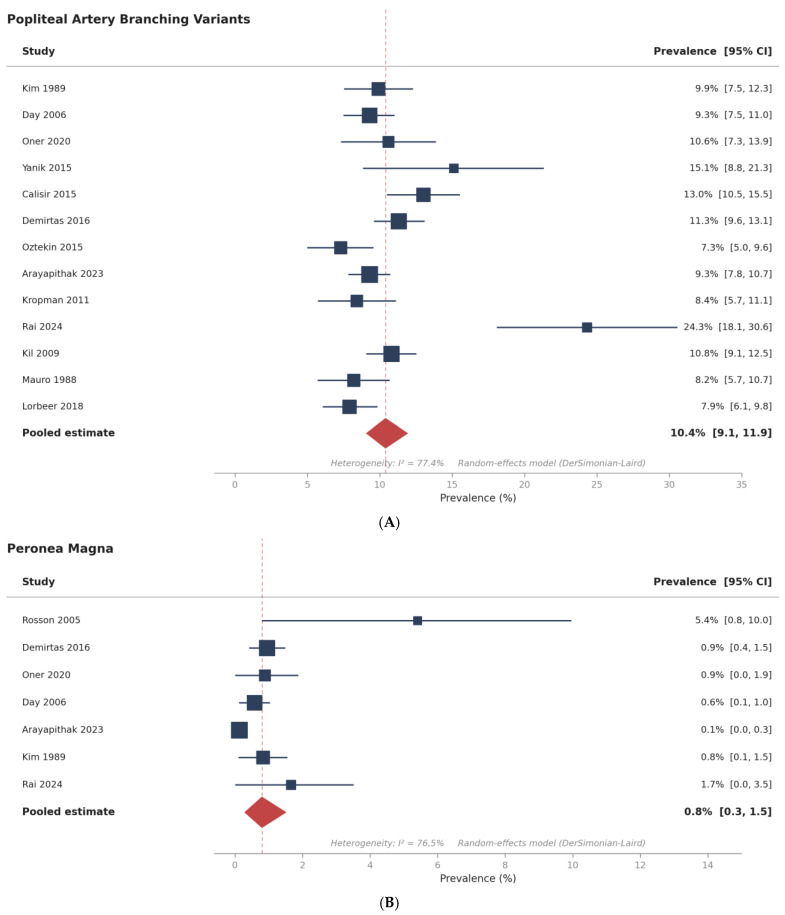
Forest plots of proportional meta-analysis for lower-limb arterial variations: (**A**) popliteal branching variants; (**B**) peronea magna.

**Figure 4:**
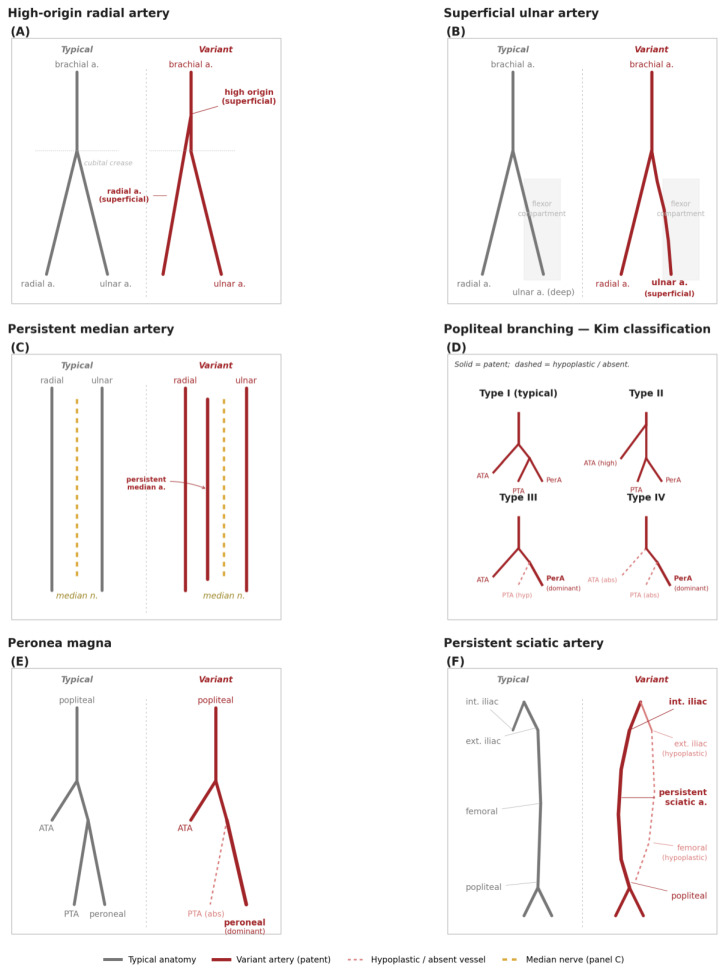
Schematic representations of the principal limb arterial variants. (**A**) High-origin radial artery: the radial artery arises proximally to the cubital crease and may pursue a superficial course; (**B**) superficial ulnar artery: the ulnar artery lies anterior to the flexor compartment, vulnerable to mistaken venous cannulation; (**C**) persistent median artery: a patent companion artery to the median nerve; (**D**) popliteal branching according to the Kim classification (Types I–IV; solid lines = patent vessels, dashed lines = hypoplastic or absent vessels); (**E**) peronea magna: the peroneal artery is the dominant or sole pedal supply, an absolute contraindication to fibula free flap harvest; (**F**) persistent sciatic artery: the embryonic axial artery persists and supplies the lower limb via the internal iliac–popliteal pathway, with hypoplasia of the femoral system (Cowie sign).

Pooled prevalence estimates and heterogeneity metrics for all six arterial variants are presented in Table 1, with forest plots shown in Figure 2 (upper-limb variants) and Figure 3 (lower-limb variants). High-origin radial artery (Figure 4A). Fifteen studies (4657 limbs) contributed data. Pooled prevalence was 11.1% (95% CI 9.2–13.1; I^2^ = 74.1%; Q = 54.08, df = 14, *p* < 0.001; prediction interval 5.0–19.0%). Haładaj and colleagues identified a characteristic “cubital crossover” morphology in 54.6% of cases [19]. Subgroup analysis suggested marginally higher prevalence on cadaveric dissection than on imaging, consistent with the higher sensitivity of direct visualization.

**Superficial ulnar artery** (Figure 4B). Eight studies (2362 limbs) contributed data. Pooled prevalence was 2.6% (95% CI 1.9–3.3; I^2^ = 0%; Q = 2.93, df = 7, *p* = 0.89). Although less common than high-origin radial artery, the variant’s superficial position renders it vulnerable to mistaken venous cannulation. The 29% amputation rate after inadvertent intra-arterial injection in 209 documented cases of upper-extremity exposure [5] establishes this variant as a high-stakes recognition target for trainees.

**Persistent median artery** (Figure 4C). Eleven studies (1507 limbs) contributed data. Pooled prevalence was 12.2% (95% CI 6.5–19.2; I^2^ = 92.6%; Q = 134.37, df = 10, *p* < 0.001). The substantial heterogeneity is largely attributable to detection methodology: studies using histological or high-resolution imaging report 30–43% [10,20], whereas macroscopic dissection typically reports 4–13% [11]. Egger’s test was not significant (*p* = 0.58), arguing against publication bias as the primary driver of variability. The methodological sensitivity itself is educationally instructive: reported prevalence depends materially on how variation is sought. Persistent median artery is associated with carpal tunnel syndrome and is relevant to forearm flap planning [21].

### 3.4. Lower-Limb Arterial Variations

Popliteal artery branching variants (Figure 4D). Thirteen studies (9174 limbs) contributed data. Pooled prevalence of any branching variant (Kim Types II and III combined) was 10.4% (95% CI 9.1–11.9; I^2^ = 77.4%; Q = 53.21, df = 12, *p* < 0.001; prediction interval 5.9–16.0%). One large multidetector CT angiography series reported approximately twofold higher variant prevalence in female compared with male subjects [22]; this observation derives from a single study and a pooled sex-stratified meta-analysis was not feasible because most other included studies did not report sex-disaggregated prevalence. The Kim classification [3] continues to provide the dominant teaching scaffold; a recent multidetector CT angiography series in 181 Indian limbs reported a high overall variant rate (24.3%), driven by Type III patterns [12].

**Peronea magna** (Figure 4E) Seven studies (5059 limbs) contributed data. Pooled prevalence was 0.8% (95% CI 0.3–1.5; I^2^ = 76.5%; Q = 25.54, df = 6, *p* < 0.001). Despite its rarity, peronea magna constitutes an absolute contraindication to fibula free flap harvest, where the peroneal artery is the dominant or sole supply to the foot. Documented cases of limb loss [6] underscore the criticality of preoperative vascular imaging and of deliberate teaching of this variant during reconstructive training.

**Persistent sciatic artery** (Figure 4F). This variant, representing retention of the embryonic axial artery, is exceedingly rare (estimated prevalence 0.03–0.06%) and was not amenable to proportional meta-analysis. A systematic review of 167 published cases reported aneurysm formation in 48% and highlighted Cowie’s sign—a palpable popliteal pulse with absent femoral pulse—as a pathognomonic clinical examination finding [23].

### 3.5. Sensitivity Analyses and Small-Study Effects

**Figure 5:**
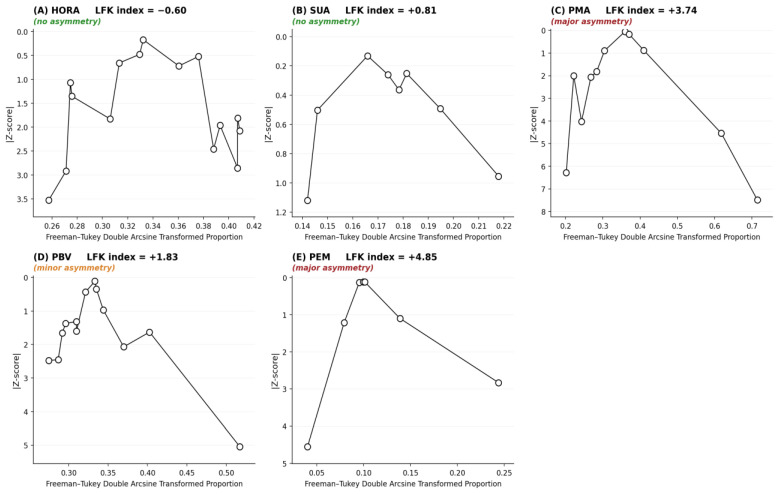
Doi plots with the LFK (Luis Furuya-Kanamori) index for each of the five proportional meta-analyses. For each study, the Freeman-Tukey double-arcsine transformed proportion (effect size) is plotted on the horizontal axis and the absolute Z-score on the vertical axis, with the vertical axis inverted so that |Z| = 0 sits at the top of each panel; studies are shown as open circles connected by thin solid lines in ascending order of effect size. LFK values are interpreted as no asymmetry (|LFK| ≤ 1), minor asymmetry (1 < |LFK| ≤ 2), or major asymmetry (|LFK| > 2); thresholds follow Furuya-Kanamori et al. (2018). Major asymmetry for persistent median artery (LFK = +3.74) and peronea magna (LFK = +4.85) indicates that smaller studies systematically reported higher prevalence than larger studies; see Section 3.5 for interpretation.

Excluding the seven low-quality studies did not materially alter pooled prevalence estimates. For high-origin radial artery, the sensitivity estimate was 11.8% (95% CI 9.4–14.4; k = 11) compared with the primary estimate of 11.1%, a difference of 0.7 percentage points. Egger’s regression suggested potential small-study effects for high-origin radial artery and popliteal branching variants but was not significant for persistent median artery (*p* = 0.58). For variants with k < 10 contributing studies, formal small-study assessment was not performed because of limited statistical power. The Doi plot analyses with the LFK index (Figure 5) gave the following results: high-origin radial artery, LFK = −0.60 (no asymmetry); superficial ulnar artery, LFK = +0.81 (no asymmetry); persistent median artery, LFK = +3.74 (major asymmetry); popliteal branching variants, LFK = +1.83 (minor asymmetry); and peronea magna, LFK = +4.85 (major asymmetry). The major-asymmetry findings for persistent median artery and peronea magna indicate that smaller studies systematically reported higher prevalence than larger studies. For persistent median artery this is the well-documented detection-sensitivity effect (smaller dedicated cadaveric series using histology or high-resolution imaging detect more median arteries than large imaging cohorts; see Section 3.6). For peronea magna the asymmetry likely reflects the small absolute number of variant cases per study (range 0–9) combined with possible publication preference for series demonstrating the variant; given the rarity of the variant, the absolute clinical message is unchanged. The LFK results are consistent in direction with the Egger’s regression assessments and, following current recommendations for prevalence meta-analyses, supersede the Egger’s results for the purpose of inferring small-study effects.

### 3.6. Exploratory Analyses of Heterogeneity

For the three syntheses with the highest heterogeneity (persistent median artery I^2^ = 92.6%, popliteal branching I^2^ = 77.4%, peronea magna I^2^ = 76.5%), the following exploratory observations were made. Persistent median artery prevalence was strongly stratified by detection methodology: studies using histological or high-resolution imaging reported 30–43%, while macroscopic cadaveric dissection reported 4–13% (Section 3.3). For popliteal branching variants, the highest single estimate (24.3%) came from a single Indian multidetector CT angiography series in 181 limbs [12], suggesting a possible regional or methodological component; with only one Indian and one Turkish series among the 13 contributing studies, a quantitative subgroup analysis by continent was not statistically informative. For peronea magna, the high I^2^ reflects between-population variation in the prevalence of dominant peroneal supply combined with the small absolute number of variant cases per study (range 0–9), which inflates between-study variance in the proportional meta-analysis even when the underlying prevalence is similar; publication period did not show a monotonic trend on exploratory inspection. We retained the random-effects pooled estimate as the headline result because no individual subgroup was sufficiently populated to displace the overall estimate, but these heterogeneity considerations are reflected in the cautious wording of the practical conclusions. Pre-specified subgroup analyses by study design and by geographic region (Table 2) were additionally performed. For high-origin radial artery, the pooled estimate was almost identical between cadaveric studies (11.18%, 95% CI 9.26–13.25%, k = 13) and imaging studies (11.69%, 95% CI 4.82–21.05%, k = 2), although the imaging subgroup was small. Persistent median artery was studied almost exclusively in cadaveric series (k = 10 of 11), preventing a meaningful design comparison; popliteal branching variants and peronea magna were both studied exclusively in imaging series and likewise could not be design-stratified. Regional analysis showed moderate variation for popliteal branching variants—Europe 8.69% (k = 3), North America 9.23% (k = 2), Asia 11.86% (k = 8)—with the higher Asian estimate driven principally by the Turkish CT-angiography cohorts and the Indian multidetector series. For persistent median artery, the regional spread was wide and reflects both methodological and population factors: Europe 4.75% (k = 2), Asia 10.73% (k = 4), Oceania 18.25% (k = 2), North America 19.38% (k = 3), with the high North-American and Oceania estimates driven by the high-resolution cadaveric series of Lucas et al. [20] and Ellis et al. [10]. We interpret these regional differences as hypothesis-generating, given uneven distribution of studies across regions and the established detection-methodology effect on reported prevalence.

## 4. An Evidence-Based Educational Framework

### 4.1. From Peripheral Footnote to Core Competency

Pooled prevalence requires a quantitative recasting of how often surgical trainees will encounter variation in practice. Combining our upper-limb estimates, in a 100-student anatomy course examining 25 cadavers (50 upper limbs), approximately 9 limbs will demonstrate at least one clinically relevant arterial variant; on a typical surgical residency operating list, encounters with popliteal branching variants will occur at a rate consistent with one in ten lower-limb vascular procedures. Variation is therefore an expected—and assessable—feature of surgical practice, not an exception. Thematic analysis of the 57 included studies generated four organizing principles for surgical education. These principles are presented as evidence-informed proposals derived from the synthesized literature; they have not been tested in controlled educational outcomes studies, and validation priorities are discussed in Section 5.2.

### 4.2. Principle 1—Embryological Reasoning as Foundation

Variation is best taught not as memorized lists but as the morphological expression of developmental channel selection from the primitive vascular plexus. The embryological frame explains both why variations occur and which configurations are anatomically plausible. Trainees who internalize this mechanism transfer the reasoning across systems—for example, recognizing the parallel logic by which a high-origin radial artery and a persistent median artery represent, respectively, proximal channel retention and failure of normal regression.

### 4.3. Principle 2—Clinical-Consequence Integration

Each variant should be paired explicitly with the clinical scenario in which recognition affects outcomes: superficial ulnar artery with intra-arterial injection and the documented 29% amputation rate [5]; peronea magna with fibula free flap planning and reported limb loss [6]; popliteal branching variants with bypass and endovascular planning [3,22]. Consequence-anchored teaching converts prevalence statistics into motivated learning.

### 4.4. Principle 3—Systematic Identification Protocols

Trainees should be drilled in structured search routines: tracing the brachial artery from axilla to cubital fossa with attention to vessels crossing the median nerve superficially, examining palmar arch completeness, evaluating the popliteal trifurcation pattern on every CT angiogram. Imaging checklists and dissection guides operationalize the principle and produce reproducible competence.

### 4.5. Principle 4—Competency-Based Assessment

Variation recognition belongs in formative and summative assessment: identification on prosected or fresh-frozen specimens; interpretation of angiographic and CT angiographic studies featuring variant anatomy; and clinical vignettes requiring trainees to anticipate variation when planning a procedure. Assessment closes the loop between the prevalence data presented above and demonstrable competency at each level of training. Practical implementations might include: (i) an OSCE imaging-interpretation station in which trainees are shown a CT angiogram featuring a high-origin radial artery or a peronea magna and are asked to identify the variant, predict the relevant procedural risk, and propose a modification to the surgical plan; (ii) a mini-CEX-style anatomy assessment on prosected specimens of upper-limb arterial variants; and (iii) pre-procedural case-based vignettes (for example, planning a fibula free flap or a radial-artery forearm flap) requiring trainees to articulate how anatomical variation could alter the surgical plan and to enumerate the imaging confirmations they would obtain. These approaches operationalise the framework’s principles within existing UME and GME assessment infrastructure rather than requiring purpose-built instruments.

### 4.6. Curricular Integration Across the Training Continuum

**Pre-clinical years (UME, Years 1–2).** Variation is introduced as expected phenotypic diversity rather than as an exception. Embryological mechanisms are paired with prevalence figures from this review to ground the abstract in measurable expectation. Dissection encounters are framed as teachable moments rather than nuisances.

**Clinical years and surgical clerkship (UME, Years 3–4).** Case-based teaching connects pre-procedural imaging interpretation to variation recognition. Emergency medicine and procedural rotations reinforce vascular access risk; reconstructive surgery rotations introduce flap-relevant variation. Variation appears explicitly on procedural skills assessments.

**Figure 6:**
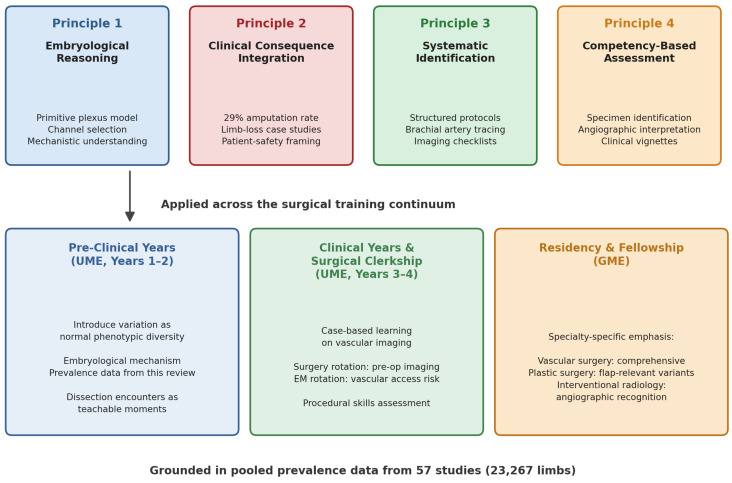
Evidence-based educational framework for integrating limb arterial variations into the surgical training continuum.

**Residency and fellowship (GME).** Specialty-specific emphasis is layered: vascular surgery trainees demand mastery across all major variants; plastic and reconstructive surgery trainees require competence with the radial artery (forearm flap) and the peroneal artery (fibula flap); interventional radiology trainees focus on angiographic recognition. The four-principle framework supplies a common language across specialties; the prevalence estimates supply a common evidence base. The four principles and their mapping across the training continuum are summarised in Figure 6.

## 5. Discussion

This systematic review and proportional meta-analysis provides, to our knowledge, the first synthesized prevalence estimates across multiple limb arterial variants designed expressly for surgical-education planning. Approximately one in nine upper limbs demonstrates a high-origin radial artery, one in ten lower limbs demonstrates a popliteal branching variant, and approximately one in eight upper limbs demonstrates a persistent median artery. These figures place variation squarely inside the band of clinical events for which surgical training is expected to prepare trainees.

The minimal heterogeneity (I^2^ = 0%) for superficial ulnar artery indicates that the approximately 2.6% prevalence is portable across populations and detection methods, supporting a shared educational message about cannulation risk. By contrast, the high heterogeneity for persistent median artery is itself an instructive finding: histological and high-resolution techniques detect substantially more median arteries than macroscopic dissection alone, with the highest reported prevalence of 42.9% in a recent dedicated study [10]. Trainees benefit from explicit discussion of how detection method shapes reported prevalence; this is a transferable methodological lesson that extends well beyond arterial anatomy.

The clinical-consequence record makes the case for treating variation recognition as a patient-safety competency. The 29% amputation rate after inadvertent intra-arterial drug injection [5] and documented limb loss after fibula harvest in unrecognized peronea magna [6] are not abstract risks; they are documented complications that better recognition would have prevented. Aligning education with these consequences requires explicit competency definition and assessment—the rationale for Principle 4.

Translating these prevalence estimates into vascular and reconstructive surgical practice clarifies the operative relevance of variation recognition. For vascular surgeons, a popliteal branching variant rate of approximately one in ten lower-limb procedures means that pre-procedural assessment of the trifurcation pattern on multidetector CT angiography should be a routine component of below-knee bypass and endovascular planning, particularly when a Kim Type III or IV configuration would alter the choice of distal target or access site. For plastic and reconstructive surgeons, the absolute contraindication that an unrecognized peronea magna poses to fibula free flap harvest [6] mandates that the dominant pedal supply be confirmed on CT or MR angiography in every preoperative work-up. For interventional and emergency clinicians performing upper-limb arterial cannulation or radial access, the 2.6% prevalence of superficial ulnar artery and the 11.1% prevalence of high-origin radial artery convert from anatomical curiosities into incident events for which procedural protocols and trainee supervision should be calibrated. Variation is therefore not a peripheral concern; it is a quantifiable component of contemporary procedural risk that informs case planning, imaging strategy, and consent.

A direct comparison of the included studies’ findings is also instructive. For high-origin radial artery, the contributing studies cluster between approximately 7% and 16%, with the notable outlier of Haładaj et al. [19], whose detailed dissection protocol yielded the highest estimate; the convergence of dissection and imaging estimates around the pooled 11.1% supports the educational claim that approximately one in nine upper limbs presents the variant. For persistent median artery, the spread between Lucas et al. [20] (cadaveric, 35.0%), Ellis et al. [10] (cadaveric, 42.9%), and the older macroscopic series (4–13%) demonstrates that the disagreement is not random error but a systematic effect of detection sensitivity; trainees who understand this stratification will interpret reported prevalence in context rather than as a fixed population property. For peronea magna, all contributing series converge near or below 1%, with the variability in the pooled estimate driven by the small absolute number of cases per study rather than disagreement about the underlying prevalence.

### 5.1. Limitations

Title and abstract screening was performed by a single reviewer (A.N.A.), and this is the most consequential methodological limitation of the present review. A single-reviewer first-pass screen may exclude relevant studies that a second reviewer would have retained; we mitigated this risk by routinely flagging uncertain records for adjudication by a second reviewer (Ş.A.-L.), by applying independent dual-reviewer full-text assessment with substantial inter-rater agreement (κ = 0.82) at the inclusion stage, and by hand-searching the reference lists of all included studies and forward-tracking citations of three landmark studies [1,2,3] to recover potentially missed records. Nevertheless, the risk of missed inclusion at the screening stage cannot be eliminated and may bias prevalence estimates if missed studies systematically differ from the included pool. Future updates of this review should adopt full dual-reviewer screening from the title and abstract stage onward. A further methodological constraint is that the persistent sciatic artery, an embryologically informative but exceedingly rare variant (estimated prevalence 0.03–0.06%), did not provide sufficient cases per included study to permit proportional meta-analysis and was therefore reported narratively; no pooled prevalence estimate is offered for this variant, and educational coverage of the persistent sciatic artery must continue to rely on narrative case series. The included studies are heterogeneous in methodology, sample composition, and quality; although the random-effects model accounts for between-study variance, residual heterogeneity for some variants indicates true variability across populations and detection methods, which the exploratory analyses in Section 3.6 attempt to characterize. Including historical studies with limited methodological reporting may introduce bias, although sensitivity analyses excluding these studies did not materially alter pooled estimates. Finally, the educational framework is evidence-informed but has not yet been empirically tested in a controlled educational study.

### 5.2. Future Directions

Priorities for future work include (1) prospective evaluation of how alternative curricular approaches affect trainee variation-recognition skills; (2) development and validation of assessment instruments for variation identification across UME and GME; (3) simulation studies examining whether deliberate practice with variant anatomy improves intraoperative performance; and (4) prevalence studies from currently underrepresented geographic regions to strengthen the evidence base used in curricular design.

## 6. Conclusions

Across 57 studies and 23,267 limbs, limb arterial variation is sufficiently common to merit deliberate curricular attention rather than incidental coverage. The four-principle framework presented here—embryological reasoning, clinical-consequence integration, systematic identification protocols, and competency-based assessment—is offered as an evidence-informed proposal for embedding variation recognition across UME, residency, and subspecialty fellowship training. Prospective educational outcome studies will be needed to determine whether deliberate curricular implementation translates into measurable gains in trainee variation-recognition skills and, ultimately, in patient outcomes.

## Figures and Tables

**Table 1:** Pooled prevalence estimates and heterogeneity metrics for the six limb arterial variants. k = number of contributing studies; PI = prediction interval; NA = Egger’s regression not performed (k < 10). † The 57 included studies provided data on multiple variants (counts are not additive across rows). Note: small-study effects assessed via Egger’s regression are tabulated here for historical reference; following current methodological recommendations for proportional meta-analyses, small-study effects are now formally assessed by the DOI plot with LFK index (Figure 5).

Variant	k	Total Limbs	Pooled Prevalence % (95% CI)	95% PI	I^2^ (%)	Q (df), *p*	Egger’s *p*
**High-origin radial artery**	15	4657	11.1 (9.2–13.1)	5.0–19.0	74.1	54.08 (14), <0.001	<0.05
**Superficial ulnar artery**	8	2362	2.6 (1.9–3.3)	—	0	2.93 (7), 0.89	NA
**Persistent median artery**	11	1507	12.2 (6.5–19.2)	—	92.6	134.37 (10), <0.001	0.58
**Popliteal branching variants**	13	9174	10.4 (9.1–11.9)	5.9–16.0	77.4	53.21 (12), <0.001	<0.05
**Peronea magna**	7	5059	0.8 (0.3–1.5)	—	76.5	25.54 (6), <0.001	NA
**Persistent sciatic artery**	—	—	narrative (est. 0.03–0.06)	—	—	—	—
**Total/overall (six variants)**	57 †	23,267	—	—	—	—	—

**Table 2:** Subgroup analyses for each variant by study design (cadaveric vs. imaging-based) and by geographic region (continent). Pooled prevalence with 95% confidence intervals is reported under the Freeman-Tukey/DerSimonian-Laird random-effects framework. Subgroups are shown only when at least two contributing studies were available within the subgroup level. Variants studied within a single design category (e.g., popliteal branching variants—all imaging) cannot be design-stratified.

Variant	Subgroup Category	Subgroup Level	k	Total Limbs	Pooled Prevalence % (95% CI)
**High-origin radial artery**	Design	Cadaveric	13	4057	11.18 (9.26–13.25)
**High-origin radial artery**	Design	Imaging	2	600	11.69 (4.82–21.05)
**High-origin radial artery**	Region	North America	3	1099	13.14 (9.48–17.30)
**High-origin radial artery**	Region	Europe	6	2266	10.31 (8.00–12.89)
**High-origin radial artery**	Region	Asia	3	670	9.92 (4.85–16.54)
**High-origin radial artery**	Region	Africa	2	122	12.05 (6.93–18.34)
**Superficial ulnar artery**	Design	Cadaveric	8	2362	2.84 (2.21–3.54)
**Superficial ulnar artery**	Region	Europe	5	1100	2.70 (1.83–3.74)
**Persistent median artery**	Design	Cadaveric	10	1374	13.10 (6.82–21.03)
**Persistent median artery**	Region	Europe	2	668	4.75 (3.20–6.58)
**Persistent median artery**	Region	Asia	4	294	10.73 (6.60–15.71)
**Persistent median artery**	Region	Oceania	2	198	18.25 (0.97–49.85)
**Persistent median artery**	Region	North America	3	347	19.38 (3.78–43.09)
**Popliteal branching variants**	Design	Imaging	13	9174	10.52 (9.17–11.95)
**Popliteal branching variants**	Region	North America	2	1069	9.23 (7.57–11.03)
**Popliteal branching variants**	Region	Europe	3	2234	8.69 (7.55–9.89)
**Popliteal branching variants**	Region	Asia	8	5871	11.86 (9.78–14.12)
**Peronea magna**	Design	Imaging	7	5059	0.96 (0.44–1.67)
**Peronea magna**	Region	North America	2	698	2.61 (0.03–9.14)
**Peronea magna**	Region	Asia	4	3324	0.78 (0.21–1.71)

## Data Availability

All data supporting the findings of this study are available within the article and its supplementary materials.

## Supplementary Materials

The following supporting information can be downloaded at https://www.mdpi.com/article/10.3390/diagnostics16142163/s1: PRISMA 2020 Checklist; Supplementary Appendix S1: Full electronic search strategy for each database; Supplementary Table S1: Characteristics of all included studies.

## References

[1] Rodríguez-Niedenführ M., Vázquez T., Nearn L., Ferreira B., Parkin I., Sañudo J.R. (2001). Variations of the arterial pattern in the upper limb revisited: A morphological and statistical study, with a review of the literature. J. Anat..

[2] McCormack L.J., Cauldwell E.W., Anson B.J. (1953). Brachial and antebrachial arterial patterns: A study of 750 extremities. Surg. Gynecol. Obstet..

[3] Kim D., Orron D.E., Skillman J.J. (1989). Surgical significance of popliteal arterial variants: A unified angiographic classification. Ann. Surg..

[4] Drake R.L., McBride J.M., Lachman N., Pawlina W. (2009). Medical education in the anatomical sciences: The winds of change continue to blow. Anat. Sci. Educ..

[5] Devulapalli C., Han K.D., Bello R.J., LaPorte D.M., Hepper C.T., Katz R.D. (2015). Inadvertent intra-arterial drug injections in the upper extremity: Systematic review. J. Hand Surg. Am..

[6] Rosson G.D., Singh N.K. (2005). Devascularizing complications of free fibula harvest: Peronea arteria magna. J. Reconstr. Microsurg..

[7] Meyer J.H.F., Land R., Rust C. (2003). Threshold concepts and troublesome knowledge: Linkages to ways of thinking and practising within the disciplines. Improving Student Learning—Theory and Practice 10 Years on.

[8] Henry B.M., Tomaszewski K.A., Walocha J.A. (2016). Methods of evidence-based anatomy: A guide to conducting systematic reviews and meta-analysis of anatomical studies. Ann. Anat..

[9] Page M.J., McKenzie J.E., Bossuyt P.M., Boutron I., Hoffmann T.C., Mulrow C.D., Shamseer L., Tetzlaff J.M., Akl E.A., Brennan S.E. (2021). The PRISMA 2020 statement: An updated guideline for reporting systematic reviews. BMJ.

[10] Ellis C., Thibault D., Lencke J., Hemric L.D. (2025). Prevalence and anatomical significance of the persistent median artery: A cadaveric study. PLoS ONE.

[11] Tran P., Bennett D., Eversole E.A., Al-Sharfeen A., Nguyen V., Frison A.C., Arguedas C., Sadacharan C., Pinkas A., Tippen S.P. (2026). Persistent median artery prevalence: A cadaveric study. Cureus.

[12] Rai A., Chopra J., Irfan A., Roy S., Gourav G., Parihar A., Kumar S. (2024). Variations in the termination of the popliteal artery: A multidetector computed tomography angiography (CTA)-based retrospective study. Cureus.

[13] Landis J.R., Koch G.G. (1977). The measurement of observer agreement for categorical data. Biometrics.

[14] Henry B.M., Tomaszewski K.A., Ramakrishnan P.K., Roy J., Vikse J., Loukas M., Tubbs R.S., Walocha J.A. (2017). Development of the Anatomical Quality Assessment (AQUA) tool for the quality assessment of anatomical studies. Clin. Anat..

[15] Freeman M.F., Tukey J.W. (1950). Transformations related to the angular and the square root. Ann. Math. Stat..

[16] Higgins J.P.T., Thompson S.G., Deeks J.J., Altman D.G. (2003). Measuring inconsistency in meta-analyses. BMJ.

[17] Egger M., Davey Smith G., Schneider M., Minder C. (1997). Bias in meta-analysis detected by a simple, graphical test. BMJ.

[18] Furuya-Kanamori L., Barendregt J.J., Doi S.A.R. (2018). A new improved graphical and quantitative method for detecting bias in meta-analysis. Int. J. Evid. Based Healthc..

[19] Haładaj R., Wysiadecki G., Dudkiewicz Z., Polguj M., Topol M. (2018). The high origin of the radial artery (brachioradial artery): Its anatomical variations, clinical significance, and contribution to the blood supply of the hand. BioMed Res. Int..

[20] Lucas T., Kumaratilake J., Henneberg M. (2020). Recently increased prevalence of the human median artery of the forearm: A microevolutionary change. J. Anat..

[21] Carry P.M., Nguyen A.K., Merritt G.R., Ciarallo C., Chatterjee D., Park J., Miller N.H., Scott F.A. (2018). Prevalence of persistent median arteries in the pediatric population on ultrasonography. J. Ultrasound Med..

[22] Oner S., Oner Z. (2020). Popliteal artery branching variations: A study on multidetector CT angiography. Sci. Rep..

[23] van Hooft I.M., Zeebregts C.J., van Sterkenburg S.M.M., de Vries W.R., Reijnen M.M.P.J. (2009). The persistent sciatic artery. Eur. J. Vasc. Endovasc. Surg..

