# Supplementary material for: Prevalence and Clinical Significance of Limb Arterial Variations: A Systematic Review and Proportional Meta-Analysis with an Evidence-Based Educational Framework
Source: Diagnostics (Basel). 2026 Jul 10;16(14):2163. doi: 10.3390/diagnostics16142163 (PMC13407564; doi:10.3390/diagnostics16142163)
Supplement: Supplementary file 1 [file diagnostics-16-02163-s001.zip › diagnostics-4363915_PRISMA_2020_Checklist.pdf]

PRISMA 2020 Checklist

Manuscript ID: diagnostics-4363915 (revised)

Title: Prevalence and Clinical Significance of Limb Arterial Variations: A Systematic Review and Proportional Meta-Analysis with an Evidence-Based Educational Framework

References to "Section X.Y", page numbers, and paragraphs correspond to the revised manuscript (Manuscript-Diagnostics-Revised.docx, 18-page PDF rendering) submitted with this checklist.

Section and Topic	Item #	Checklist item	Location where item is reported
**TITLE**			
Title	1	Identify the report as a systematic review.	Title page, Page 1 — the title explicitly contains "A Systematic Review and Proportional Meta-Analysis."
**ABSTRACT**			
Abstract	2	See the PRISMA 2020 for Abstracts checklist.	Page 1, Abstract (single paragraph). The abstract reports objectives, eligibility criteria, information sources, methods of synthesis, results with confidence intervals, and conclusions.
**INTRODUCTION**			
Rationale	3	Describe the rationale for the review in the context of existing knowledge.	Page 1, Section 1 (Introduction), paragraphs 1–3.
Objectives	4	Provide an explicit statement of the objective(s) or question(s) the review addresses.	Pages 1–2, Section 1 (Introduction), final paragraph (objectives 1–3).
**METHODS**			
Eligibility criteria	5	Specify the inclusion and exclusion criteria for the review and how studies were grouped for the syntheses.	Page 2, Section 2.2 (Eligibility Criteria), single paragraph (PICOS framework).
Information sources	6	Specify all databases, registers, websites, organisations, reference lists and other sources searched or consulted to identify studies. Specify the date when each source was last searched or consulted.	Page 3, Section 2.3 (Information Sources and Search Strategy), paragraph 1 (six databases searched from inception through December 2024; chronology of three online-ahead-of-print references clarified in the revised version).
Search strategy	7	Present the full search strategies for all databases, registers and websites, including any filters and limits used.	Page 3, Section 2.3 (summary); full strategy provided in Supplementary Appendix S1.
Selection process	8	Specify the methods used to decide whether a study met the inclusion criteria of the review, including how many reviewers screened each record and each report retrieved, whether they worked independently, and if applicable, details of automation tools used in the process.	Page 3, Section 2.4 (Study Selection and Data Extraction) (dual-reviewer independent full-text screening; κ for inter-rater agreement; no automation tools used).
Data collection process	9	Specify the methods used to collect data from reports, including how many reviewers collected data from each report, whether they worked independently, any processes for obtaining or confirming data from study investigators, and if applicable, details of automation tools used.	Page 3, Section 2.4 (Study Selection and Data Extraction), final sentences (primary extraction by A.N.A. with independent verification by Ş.A.-L.; no automation tools).
Data items	10a	List and define all outcomes for which data were sought. Specify whether all results that were compatible with each outcome domain in each study were sought (e.g. for all measures, time points, analyses), and if not, the methods used to decide which results to collect.	Page 3, Section 2.4 — primary outcomes: prevalence of arterial variants per limb; all reported prevalence estimates per variant per study were collected.

Data items	10b	List and define all other variables for which data were sought (e.g. participant and intervention characteristics, funding sources). Describe any assumptions made about any missing or unclear information.	Page 3, Section 2.4 — extracted variables listed: study identifiers, design, detection method, sample size in limbs, variant type, count of variants, prevalence, reported clinical consequences.
Study risk of bias assessment	11	Specify the methods used to assess risk of bias in the included studies, including details of the tool(s) used, how many reviewers assessed each study and whether they worked independently, and if applicable, details of automation tools used in the process.	Page 3, Section 2.5 (Quality Assessment) — Anatomical Quality Assessment (AQUA) tool, dual-reviewer independent appraisal, inter-rater κ reported.
Effect measures	12	Specify for each outcome the effect measure(s) (e.g. risk ratio, mean difference) used in the synthesis or presentation of results.	Page 3, Section 2.6 (Statistical Analysis), paragraph 1 — pooled prevalence as proportion with 95% confidence intervals and 95% prediction intervals.
Synthesis methods	13a	Describe the processes used to decide which studies were eligible for each synthesis (e.g. tabulating the study intervention characteristics and comparing against the planned groups for each synthesis (item 5)).	Page 3, Section 2.6 — each variant type analysed as a separate synthesis; eligibility for each synthesis matched eligibility criteria (Section 2.2).
Synthesis methods	13b	Describe any methods required to prepare the data for presentation or synthesis, such as handling of missing summary statistics, or data conversions.	Page 3, Section 2.6 — Freeman-Tukey double arcsine transformation applied to stabilise the variance of proportions.
Synthesis methods	13c	Describe any methods used to tabulate or visually display results of individual studies and syntheses.	Page 3, Section 2.6 — forest plots; presented as Figures 2 (upper limb) and 3 (lower limb).
Synthesis methods	13d	Describe any methods used to synthesize results and provide a rationale for the choice(s). If meta-analysis was performed, describe the model(s), method(s) to identify the presence and extent of statistical heterogeneity, and software package(s) used.	Page 3, Section 2.6 — DerSimonian-Laird random-effects proportional meta-analysis; heterogeneity quantified with Cochran's Q (significance threshold $p < 0.10$) and I^2 (low ≤ 25%, moderate 26–75%, high > 75%).
Synthesis methods	13e	Describe any methods used to explore possible causes of heterogeneity among study results (e.g. subgroup analysis, meta-regression).	Page 3, Section 2.6 (pre-specified subgroup approach by detection modality, continent, and publication period); Page 10, Section 3.6 NEW (Exploratory Analyses of Heterogeneity) — quantitative exploration of detection methodology, regional and temporal contributions to heterogeneity for the three high-I^2 syntheses; Pages 6–7, Section 3.3 (detection-method effect on persistent median artery prevalence).
Synthesis methods	13f	Describe any sensitivity analyses conducted to assess robustness of the synthesized results.	Page 3, Section 2.6 — sensitivity analyses excluding AQUA low-quality studies; results presented Page 10, Section 3.5.
Reporting bias assessment	14	Describe any methods used to assess risk of bias due to missing results in a synthesis (arising from reporting biases).	Page 3, Section 2.6 — Egger's regression for small-study effects performed where $k \geq 10$; results in Page 10, Section 3.5.
Certainty assessment	15	Describe any methods used to assess certainty (or confidence) in the body of evidence for an outcome.	Page 3, Section 2.5 — study-level methodological quality appraised with the AQUA tool; a formal GRADE assessment was not performed for these prevalence outcomes, as anatomical-prevalence syntheses are not well-suited to the GRADE framework (no formal prognostic, diagnostic, or interventional effect estimate).
**RESULTS**			
Study selection	16a	Describe the results of the search and selection	Page 4, Section 3.1 (Study Selection and

		process, from the number of records identified in the search to the number of studies included in the review, ideally using a flow diagram.	Characteristics) and Figure 1 (PRISMA 2020 flow diagram, Page 5).
Study selection	16b	Cite studies that might appear to meet the inclusion criteria, but which were excluded, and explain why they were excluded.	Page 5, Figure 1 (full-text exclusions and reasons enumerated in the flow diagram); Page 4, Section 3.1 added comment on the inclusion funnel from 2,847 records to 57 studies.
Study characteristics	17	Cite each included study and present its characteristics.	Page 4, Section 3.1 (summary); Pages 5–6, Table 1 NEW (per-variant k, total limbs, pooled prevalence, CI, PI, I^2, Q, Egger's p); and Supplementary Table S1 (complete study-level characteristics for all 57 included studies).
Risk of bias in studies	18	Present assessments of risk of bias for each included study.	Page 5, Section 3.2 (Quality Assessment, aggregate AQUA results) and Supplementary Table S1 (per-study AQUA ratings).
Results of individual studies	19	For all outcomes, present, for each study: (a) summary statistics for each group (where appropriate) and (b) an effect estimate and its precision (e.g. confidence/credible interval), ideally using structured tables or plots.	Pages 6–9, Sections 3.3 and 3.4; Figures 2 (upper limb forest plots, Page 8) and 3 (lower limb forest plots, Page 10) display per-study sample sizes, prevalence, 95% CIs, and weights.
Results of syntheses	20a	For each synthesis, briefly summarise the characteristics and risk of bias among contributing studies.	Pages 6–9, Sections 3.3 and 3.4; Table 1 (Pages 5–6) — number of studies (k) and limbs contributing to each pooled estimate stated per variant.
Results of syntheses	20b	Present results of all statistical syntheses conducted. If meta-analysis was done, present for each the summary estimate and its precision (e.g. confidence/credible interval) and measures of statistical heterogeneity.	Pages 6–9, Sections 3.3 and 3.4 and Table 1 (Pages 5–6) — pooled prevalence, 95% CI, I^2, Cochran's Q, df, p-value, and (where applicable) prediction intervals reported for each variant.
Results of syntheses	20c	Present results of all investigations of possible causes of heterogeneity among study results.	Page 10, Section 3.6 NEW (Exploratory Analyses of Heterogeneity) — detection methodology, regional, and temporal contributions to heterogeneity for persistent median artery, popliteal branching variants, and peronea magna; Pages 6–7, Section 3.3 (detection-method effect on persistent median artery; subgroup observation for high-origin radial).
Results of syntheses	20d	Present results of all sensitivity analyses conducted to assess the robustness of the synthesized results.	Page 10, Section 3.5 (Sensitivity Analyses and Small-Study Effects) — sensitivity estimate for high-origin radial artery (11.8% vs primary 11.1%) and qualitative statement of robustness for other variants.
Reporting biases	21	Present assessments of risk of bias due to missing results (arising from reporting biases) for each synthesis assessed.	Page 10, Section 3.5 — Egger's regression results for high-origin radial artery, popliteal branching, and persistent median artery ($p = 0.58$); Egger's p reported for each variant in Table 1 (Pages 5–6).
Certainty of evidence	22	Present assessments of certainty (or confidence) in the body of evidence for each outcome assessed.	Page 5, Section 3.2 (proportions of high, moderate, and low quality across the evidence base); per-study AQUA ratings in Supplementary Table S1. GRADE was not applied (rationale, Item 15).
**DISCUSSION**			
Discussion	23a	Provide a general interpretation of the results in the context of other evidence.	Pages 13–14, Section 5 (Discussion), paragraphs 1–5; revised manuscript adds two new Discussion paragraphs on vascular-surgical impact and direct cross-study comparison.

Discussion	23b	Discuss any limitations of the evidence included in the review.	Page 16, Section 5.1 (Limitations) — heterogeneity, methodological variability, historical studies; substantially expanded in revised manuscript to include narrative-only reporting of the persistent sciatic artery.
Discussion	23c	Discuss any limitations of the review processes used.	Page 16, Section 5.1 (Limitations), first paragraph — single-reviewer title/abstract screening identified as the most consequential methodological limitation (mitigated by dual-reviewer independent full-text assessment with substantial κ).
Discussion	23d	Discuss implications of the results for practice, policy, and future research.	Pages 11–12, Section 4 (Evidence-Based Educational Framework) — implications for practice and curricular policy; Pages 13–14, new Discussion paragraphs on vascular-surgical impact and direct cross-study comparison; Page 16, Section 5.2 (Future Directions) — research priorities.
**OTHER INFORMATION**			
Registration and protocol	24a	Provide registration information for the review, including the register name and registration number, or state that the review was not registered.	The review was not prospectively registered (e.g., in PROSPERO).
Registration and protocol	24b	Indicate where the review protocol can be accessed, or state that a protocol was not prepared.	No formal written protocol was prepared. The eligibility criteria, search strategy, data-extraction template, quality-assessment procedure, and statistical analysis plan stated in Sections 2.2–2.6 reflect the methods agreed by the review team prior to study screening and data extraction.
Registration and protocol	24c	Describe and explain any amendments to information provided at registration or in the protocol.	Not applicable — the review was not registered and no protocol amendments were made.
Support	25	Describe sources of financial or non-financial support for the review, and the role of the funders or sponsors in the review.	Page 17, Funding section — “This research received no external funding.”
Competing interests	26	Declare any competing interests of review authors.	Page 17, Conflicts of Interest section — “The authors declare no conflicts of interest.”
Availability of data, code, and other materials	27	Report which of the following are publicly available and where they can be found: template data collection forms; data extracted from included studies; data used for all analyses; analytic code; any other materials used in the review.	Page 17, Data Availability Statement; complete extracted dataset for all 57 included studies provided in Supplementary Table S1. Data collection templates and analytic code are available from the corresponding author on reasonable request.

Reference: Page MJ, McKenzie JE, Bossuyt PM, et al. The PRISMA 2020 statement: an updated guideline for reporting systematic reviews. BMJ. 2021;372:n71.
