# Supplementary material for: Prevalence and Clinical Significance of Limb Arterial Variations: A Systematic Review and Proportional Meta-Analysis with an Evidence-Based Educational Framework
Source: Diagnostics (Basel). 2026 Jul 10;16(14):2163. doi: 10.3390/diagnostics16142163 (PMC13407564; doi:10.3390/diagnostics16142163)
Supplement: Supplementary file 1 [file diagnostics-16-02163-s001.zip › Supplementary_Appendix_S1.pdf]

Supplementary Appendix S1

Full Electronic Search Strategy

Search dates: Database inception through 31 December 2024

No language or date restrictions applied during search

1. PubMed/MEDLINE

Upper limb search:

```
("radial artery"[MeSH] OR "ulnar artery"[MeSH] OR "brachial artery"[MeSH])
AND ("anatomic variation"[MeSH] OR "congenital abnormalities"[MeSH]
OR variation* OR variant* OR anomal* OR aberrant OR "high origin"
OR superficial OR persistent OR accessory)

OR "superficial ulnar artery"[tiab]
OR "high origin radial artery"[tiab] OR "brachioradial artery"[tiab]
OR "persistent median artery"[tiab]
OR "upper limb arterial variation"[tiab]
OR "superficial brachial artery"[tiab]
```

Lower limb search:

```
("popliteal artery"[MeSH] OR "tibial arteries"[MeSH]
OR "peroneal artery"[tiab] OR "fibular artery"[tiab])
AND (variation* OR variant* OR anomal* OR aberrant
OR "high division" OR branching OR classification)

OR "peronea magna"[tiab]
OR "persistent sciatic artery"[tiab]
OR "popliteal artery variation"[tiab]
OR "lower limb arterial variation"[tiab]
```

Yield: 745 records

2. Scopus

```
TITLE-ABS-KEY("radial artery" OR "ulnar artery" OR "brachial artery"
OR "popliteal artery" OR "tibial artery" OR "peroneal artery")
AND TITLE-ABS-KEY(variation OR variant OR anomaly OR aberrant
OR "high origin" OR superficial OR persistent)
AND TITLE-ABS-KEY(prevalence OR incidence OR frequency
OR cadaver* OR dissection OR angiograph* OR "CT angiography")

OR TITLE-ABS-KEY("superficial ulnar artery" OR "brachioradial artery"
OR "persistent median artery" OR "peronea magna"
OR "persistent sciatic artery")
```

Yield: 612 records

3. Web of Science

```
TS=("radial artery" OR "ulnar artery" OR "brachial artery"
OR "popliteal artery" OR "peroneal artery")
```

```
AND TS=(variation* OR variant* OR anomal* OR aberrant
OR "high origin" OR superficial OR persistent)
AND TS=(prevalence OR cadaver* OR dissection OR angiograph*)
```

Yield: 528 records

4. Embase

```
'radial artery'/exp OR 'ulnar artery'/exp OR 'brachial artery'/exp
OR 'popliteal artery'/exp
AND ('congenital blood vessel malformation'/exp
OR variation*:ti,ab OR variant*:ti,ab OR anomal*:ti,ab)
AND (prevalence:ti,ab OR cadaver*:ti,ab OR dissection:ti,ab
OR 'computed tomographic angiography':ti,ab)

OR 'superficial ulnar artery':ti,ab
OR 'persistent median artery':ti,ab OR 'peronea magna':ti,ab
```

Yield: 489 records

5. CINAHL

```
(MH "Radial Artery" OR MH "Ulnar Artery" OR MH "Brachial Artery"
OR MH "Popliteal Artery")
AND (TI variation* OR AB variation* OR TI variant*
OR AB anomal* OR TI "high origin")
```

Yield: 198 records

6. Google Scholar

Searches performed using the following query strings (first 200 results reviewed for each):

```
"upper limb" "arterial variation" prevalence cadaveric
"superficial ulnar artery" prevalence
"high origin radial artery" OR "brachioradial artery" prevalence
"persistent median artery" prevalence
"popliteal artery" variation branching prevalence
"peronea magna" prevalence
"persistent sciatic artery" prevalence
```

Yield: 275 records (after de-duplication against database results)

7. Additional Sources

Reference list searching: Reference lists of all 57 included studies and 3 relevant systematic reviews were manually screened. Yield: 35 additional records.

Citation tracking: Forward citation tracking was performed for key studies (McCormack 1953, Rodriguez-Niedenführ 2001, Kim 1989). Yield: 7 additional records.

Total records identified: 2,847 (databases) + 42 (other sources) = 2,889
